# OTUB2 Regulates YAP1/TAZ to Promotes the Progression of Esophageal Squamous Cell Carcinoma

**DOI:** 10.1186/s12575-022-00169-9

**Published:** 2022-07-18

**Authors:** Li Liu, Hu Cheng, Min Ji, Liping Su, Ziyang Lu, Xiayun Hu, Yaling Guan, Jinling Xiao, Lijuan Ma, Wei Zhang, Hongwei Pu

**Affiliations:** 1 Department of Pathology, First Affliated Hospital, Xinjiang Medical University, No. 137, Liyushan South Road, Urumqi, 830054 Xinjiang China; 2 Department of Anesthesiology, First Affliated Hospital, Xinjiang Medical University, Urumqi, Xinjiang People’s Republic of China; 3 School of Basic Medicine, Xinjiang Medical University, Urumqi, Xinjiang People’s Republic of China; 4 Qilu Hospital, Jinan, Shandong Province China; 5 Shanghai Changhai Hospital, Shanghai, China; 6 Department of Discipline Construction, First Affliated Hospital, Xinjiang Medical University, No. 137, Liyushan South Road, Urumqi, 830054 Xinjiang China

**Keywords:** Esophageal squamous cell carcinoma, OTUB2, YAP1, TAZ, Biomarker

## Abstract

**Objective:**

The effects of Otubain-2 (OTUB2) on the proliferation, invasion, and migration of esophageal squamous cell carcinoma (ESCC) were investigated by interfering with OTUB2 expression.

**Methods:**

Bioinformatics analysis was used to analyze OTUB2 expression in esophageal carcinoma and interactions between OTUB2 and YAP1/TAZ. Paraffin-embedded ESCC tissues (*n* = 183) were selected for immunohistochemical staining to detect OTUB2, YAP1, TAZ, CTGF and their relationship with clinicopathological parameters, then the survival prognosis of ESCC patients was analyzed. Immunofluorescence, western blotting, and qRT-PCR were used to evaluate OTUB2 in ESCC cell lines. Cell lines with the highest expression of OTUB2 were transfected with lentivirus to knockdown OTUB2 levels. Changes in KYSE150 cell proliferation, migration, and invasion were measured using CCK-8, wound healing, and clone formation assays. The Transwell test and flow cytometry identified OTUB2 targets and explored roles and mechanisms involved in ESCC. Effects of OTUB2 on YAP1/TAZ signaling were also observed.

**Results:**

Bioinformatics analysis revealed OTUB2 was highly expressed in esophageal cancer and was associated with YAP1/TAZ. Immunohistochemistry showed that OTUB2 expression was increased in ESCC samples compared to parcancerous tissue. YAP1 and TAZ were higher expression in ESCC tissues, mainly localized in the nucleus. Compared with controls, the proliferation, migration, and invasion ability of KYSE150 cells after OTUB2 knockdown were significantly reduced (*P* < 0.05). The protein expression levels of YAP1, TAZ and CTGF decreased after knocking down the expression of OTUB2 (*P* < 0.05). OTUB2 knockdown in ESCC cell lines suppressed YAP1/TAZ signaling.

**Conclusions:**

OTUB2 regulated the protein expression of YAP1/TAZ to promote cell proliferation, migration, invasion, and tumor development. Therefore, OTUB2 may represent a biomarker for ESCC and a potential target for ESCC treatment.

**Supplementary Information:**

The online version contains supplementary material available at 10.1186/s12575-022-00169-9.

## Introduction

Esophageal carcinoma is a common gastrointestinal malignancy, ranking eighth in the global incidence of cancer, and sixth among the causes of cancer, the most common histological subtype is esophageal squamous cell carcinoma (ESCC) [1, 2]. Because the early symptoms of patients with esophageal cancer are atypical and the clinical treatment effects are poor, it is necessary to study the proliferation, invasion, and metastasis mechanisms involved in esophageal squamous cancer, and to identify new tumor markers and treatment targets. OTUB2 is a dihydroubiquitase, a member of the ovarian tumor domain (OTU) family with effects on various biological activities of the body [3, 4]. Some researchers have found that OTUB2 promotes the development of breast cancer [5], non-small cell lung cancer [6], thyroid papilloma cancer [7], endometrial cancer [8], and liver cancer [9]. Yes-associated protein (YAP1) and transcriptional co-activator with PDZ-binding motif (TAZ) are core factors located downstream of the Hippo signaling pathway. As the main signaling targets in the Hippo signaling pathway, they participate in tumor development and in the development of ESCC [10], laryngeal squamous cancer [11], gastric cancer [12], and breast cancer [13]. Connective tissue growth factor (CTGF) is the downstream target gene of YAP1/TAZ. When the activity of YAP1/TAZ increases, its expression can be promoted. The expression level of CTGF is closely related to the occurrence and progression of various malignant tumors, angiogenesis, invasion and metastasis, and the prognosis of patients [14].

Existing studies have dedicated less effort on investigating mechanisms associated with OTUB2, YAP1 and TAZ in ESCC, and studies have shown that in breast cancer [5],OTUB2 can stabilize and activate YAP1/TAZ. By removing the polyubiquitination chain of YAP1/TAZ promotes the proliferation, invasion and migration of tumors. Thus, the question of whether OTUB2 also plays a tumor-promoting role in ESCC has also been raised. Are these processes associated with the activation of YAP1/TAZ? The purpose of this study was to explore the expression of OTUB2 in ESCC and its impact on ESCC proliferation, migration, and invasion, and to clarify its role in ESCC development. Our findings are expected to provide new targets and a rationale for the clinical treatment of ESCC.

## Materials and Methods

### Patients and Tissue Specimens

Between January 2008 and December 2018, a total of 183 paired tissue samples including ESCC tissues and corresponding adjacent normal tissues were obtained from newly diagnosed patients who received no chemotherapy or radiotherapy, at the Department of Pathology, of the First Affiliated Hospital of Xinjiang Medical University, Urumqi, Xinjiang, PRChina. The study protocol was performed under the approval of the Ethics Committee of The First Affiliated Hospital of Xinjiang Medical University. Inclusion criteria were confirmed ESCC diagnosis and no previous chemotherapy or radiotherapy, and availability of complete clinicopathological. Exclusion criteria included other cases presenting tumor metastasis and cases in which the results of immunohistochemical staining were inconclusive due to detachment were excluded. Patient prognostic related factors were analyzed using telephone follow-up to record patient survival, and OS (overall survival, OS) was defined as the time from first diagnosis of ESCC to death or final follow-up until December 31,2019.

### Bioinformatics Analysis

TIMER database (http://timer.cistrome.org) and the Gene Expression Profiling Interactive Analysis (GEPIA) database (http://gepia.cancer-pku.cn/) were used to search the expression of OTUB2 in esophageal cancer; the GeneMANIA database (http://genemania.org/) was used to search the interaction between OTUB2 and YAP1/TAZ (WWTR1:TAZ also known as WWTR1).

### Immunohistochemistry

Anti-OTUB2 (bs6236R) purchased from Bioss Reagents Co., Ltd.; anti-YAP1 (ab52771), anti-TAZ (ab242313) were purchased from the abcam Company (UK). and Anti-CTGF (23936–1-AP) were purchased from the ProteinTech, Antibody dilution concentrations were 1:50. PBS, citric acid antigen repair solution, goat serum, hydrogen peroxide, generic type II resistance were all purchased from Bioss Reagent Co., Ltd. The specific experimental protocols were as follows: after processing, which included dewaxing, antigen retrieval, and H_2_O_2_ treatment, the paraffin-embedded tissue slides were incubated with OTUB2, YAP1 TAZ and CTGF antibody overnight at 4 °C. Then, the slides were washed with phosphate buffered saline (PBS) and incubated with a biocatalytic secondary antibody. Finally, the tissue sections were treated with DAB and were then counterstained with hematoxylin. The immunoreactions were evaluated independently by two pathologists. The score was calculated based on the staining intensity and the percentage of positive cells, the staining intensity was divided into non-staining (colorless), weak-staining (light yellow), moderate-staining (light brown), and strong-staining (dark brown), and the scores were 0, 1, 2, and 3, respectively. The scoring of positive cells was as follows: 0 for 0, 1 for 0–30%, 2 for 30–60%, and 3 for 60–90%. According to the staining index SI, the staining results were scored as negative 0–3 points and positive 4–9 points, of which 4–5 points indicated low expression, 6–7 points indicated moderate expression, and 8–9 points indicated high expression. SI = percentage of positive cells × dye intensity.

### Cell Culture and Transfection

Human ESCC cell lines KYSE30, KYSE150, and KYSE450, and the human esophageal epithelial cell line SHEE, were purchased from the Shanghai Cell Bank (Shanghai, China). All cell lines were grown in DMEM (Gibco), supplemented with 10% fetal bovine serum (BI), and incubated at 37 °C in a humidified incubator containing 5% CO_2_. Specific small interfering RNA (siRNA) for OTUB2, and control siRNA were synthesized by Shanghai Genechem Co., Ltd. We first used western blotting to detect OTUB2 expression in three ESCC lines, the cell lines with the highest expression of OTUB2 were selected for lentivirus transfection to knockdown OTUB2, and the sequences with the highest knockdown efficiency were selected for subsequent molecular experiments and cell function studies.

Target sequence of shRNA:

**Table:** 

Name	Sequences
shNC	5′-TTCTCCGAACGTGTCACGT-3’
shOTUB2#1	5′-TCCCACTACAACATCCTTT-3’
shOTUB2#2	5′-AGATGGATACCGCCCTGAA-3’
shOTUB2#3	5′-TGGTGGAACTGGTAGAGAA-3’

### Western Blotting

Briefly, the ESCC cell line was digested with trypsin and washed with PBS three times. The cell lysate was added to ice at 4 °C for 20 min, then centrifugation and collected the supernatant. BCA protein quantification was used. Next, the protein samples were separated by 10% SDS PAGE (40 μg/lane), and electrophoretically transferred to polyvinylidene difluoride membranes (PVDF). Subsequently, 5% skim milk was used to seal the membrane for 2 h, and the corresponding primary antibody was incubated overnight at 4 °C. TBST was used to wash membranes three times, and the corresponding secondary antibody was added and incubated at room temperature for 1 h. Protein expression was detected by ECL chemiluminescence.

### Quantitative Reverse Transcription Polymerase Chain Reaction (qRT-PCR)

Total RNA of ESCC cells was extracted using TRIzol reagent according to the manufacturer’s instructions. Subsequently, 1 μg RNA was used to synthesize cDNA in accordance with the manufacturer’s instructions. The reaction was terminated at 85 °C for 5 s and then at 37 °C for 30 min. RT qPCR reaction was carried out according to the “two-step” procedure. (TRIzol Reagent, PrimeScriptTM one stEP RT-PCR kit, purchased by Invitrogen; QuantiNova SYBR Green PCR Kit from QIAGEN, Germany). The primer sequences used are listed as follows:

**Table:** 

Primer	Sequences
GAPDH	Forward: 5′-GAAGGTGAAGGTCGGAGTC − 3′
	Reverse: 5′-GAAGATGGTGATGGGATTTC −3′
OTUB2	Forward: 5′-AACATCCTTTATGCAGCCGAT-3′
	Reverse: 5′-GCTGCACATTTTCTTGCTACAGT-3′
YAP1	Forward: 5′-CTCACAGCAGAACCGTTTCCC-3′
	Reverse: 5′-AGCCAAAACAGACTCCATG-3′
TAZ	Forward: 5′-TCCCAGCCAAATCTCGTGATG-3′
	Reverse: 5′-AGCGCATTGGGCATACTCAT-3′
CTGF	Forward: 5′-TGTGCACCGCCAAAGATGGT-3′
	Reverse: 5′-TCTTCCAGTCGGTAAGCCGC-3′

### Immunofluorescence

ESCC and SHEE cells were seeded in a confocal laser dish for 24 h and then fixed at 4 °C for 30 minutes with 4% tissue cell fixative. After washes with 0.5% Triton X-100/PBST, cells were blocked with 5% BSA for 30 min at room temperature and incubated with OTUB2 antibody (1:300) at 4 °C overnight. Slides were then incubated with fluorescent secondary antibody (1:500) for 2 h, and then incubated with DAPI at room temperature for 10 minutes. Finally, images were taken under an inverted fluorescence microscope.

### CCK-8 Assay

Cell growth was monitored using a CCK-8 kit (Boiss Reagents Co., Ltd). When the cells grew to logarithmic phase, the cells were digested with trypsin to prepare cell suspensions and counted. They were divided into the KYSE150 group, KYSE150 sh-non group (shNC), and KYSE150 shOTUB2 group (shOTUB2#1). Briefly, cells were plated at 5000 cells/well in 96-well plates and cultured for 72 h. After every 24 h, the 10 μL CCK-8 reagent was added and the optical density value at 450 nm for each well was obtained using a microplate reader. The CCK-8 assay was performed for three times in triplicate.

### Plate Clone Formation Assay

ESCC cells were seeded in 6-well plates at the density of 1000 cells/well. After continuous culture for 7 days, the cell clones were fixed with 4% tissue cell fixative and incubated with crystal violet. Clones containing > 50 cells were counted under an inverse microscope. The plate clone formation assay was performed three times in triplicate.

### Flow Cytometry

ESCC cells (5 × 10^5^/well) were seeded into 6-well plates and cultured for 48 h at 37 °C. After the ESCC cells were collected, cells were stained with an Annexin V-FITC apoptosis detection kit (BD Biosciences, USA) and flow cytometry was used for apoptosis detection.

### Wound Healing Assay

After transfection for 48 h, the ESCC cells were seeded into a six-well plate at a density of 1.0 × 10^6^ cells/mL until the confluence reached 90%. A 20-μL pipette tip was used to draw a straight-line wound. The cell debris then washed with PBS and they were cultured continuously in medium. The wound areas and migrated cells in each group were assessed after 24 h and 48 h.

### Transwell Assay

Transwell assays were performed following the manufacturer’s instructions to analyze the invasive ability of ESCC cells. A Transwell plate with 8-mm pore size chambers precoated with Matrigel (Corning Incorporated, Corning, NY, USA) were used. In brief, the number of cells per well in the Transwell chamber was 1 × 10^5^ and 500 μL complete culture medium containing 10% FBS was added to each lower chamber of the Transwell dish. Cell suspension was added to the upper chamber and placed in the cell incubator for 24 h. Next, the medium was removed and the cells were fixed with methanol and stained with 500 μL of 0.1% crystal violet solution and allowed to stain cells at room temperature for 10 min. The cells which failed to migrate or invade the Matrigel were removed with a swab, then the Migrated and invasive cells were pictured and their numbers calculated from five randomly selected microscopic fields.

### Statistical Analysis

All experiments were performed in triplicate. Statistical analysis was conducted using SPSS version 22.0 software and GraphPad prism version 8. For comparisons between two groups, a Student’s *t* test or chi-squared test was used. *P* < 0.05 were considered to be statistically significant.

## Results

### Bioinformatics Analysis Was Used Analyzed the Expression of OTUB2 in Esophageal cancer and its Relationship with YAP1/TAZ

**Fig. 1:**
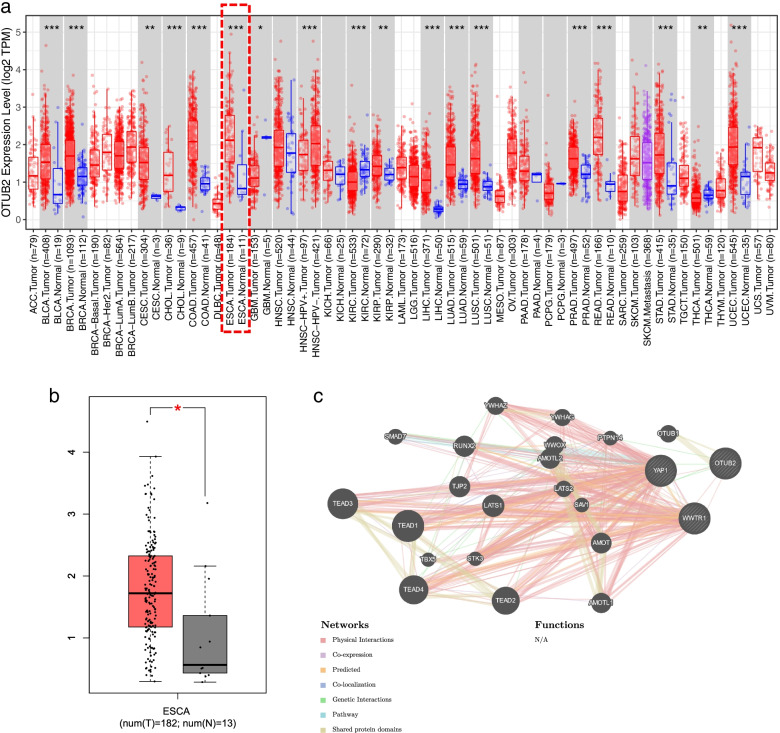
Bioinformatics analysis of the expression of OTUB2 in esophageal carcinoma and its relationship with YAP1/TAZ (WWTR1). **A** Expression of OTUB2 in various tumors and corresponding adjacent tissues; **B** Expression of OTUB2 in esophageal cancer and normal tissues; **C** The interaction relationship between OTUB2 and YAP1 / TAZ (WWTR1)

Following TIMER and GEPIA database analysis OTUB2 expression in various tumors, revealed that OTUB2 was highly expressed in various tumor tissues, and the expression in esophageal cancer tissues was significantly higher than that in normal esophageal epithelial tissues (Fig. 1A, B). Searching OTUB2 and YAP1/TAZ (WWTR1) in the GeneMANIA database revealed that there was interaction between them (Fig. 1C).

### Expression of OTUB2 and YAP1/TAZ in ESCC Tissues and its Relationship with Clinicopathological Parameters

**Fig. 2:**
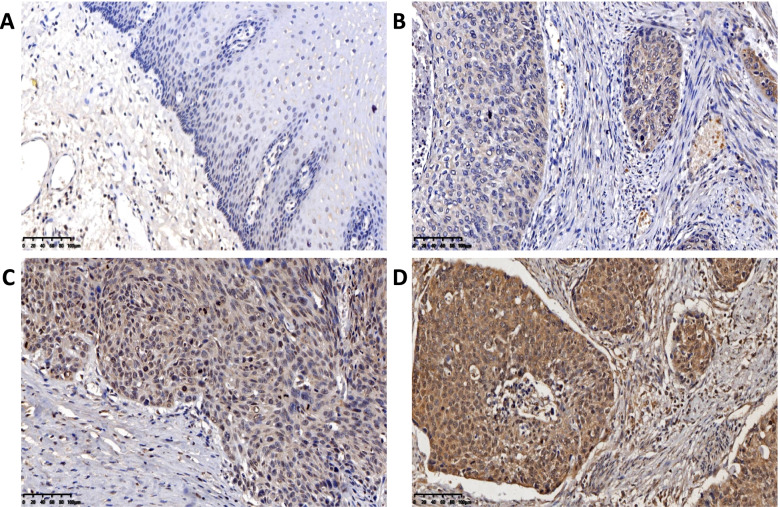
Immunohistochemical results of clinical ESCC paraffin specimens. **A** OTUB2 expression is negative in normal adjacent tissues esophageal to cancer tissue; **B** OTUB2 shows low expressed in ESCC; **C** OTUB2 is moderately expressed in ESCC; **D** OTUB2 is highly expressed in ESCC

**Fig. 3:**
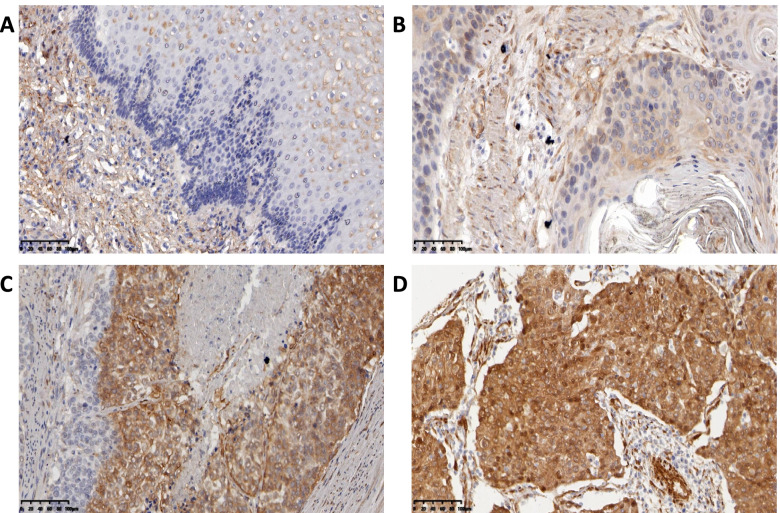
Immunohistochemical results of clinical ESCC paraffin specimens. **A** YAP1 expression is negative in normal esophageal tissues; **B** YAP1 shows low expressed in ESCC; **C** YAP1 is moderately expressed in ESCC; **D** YAP1 is highly expressed in ESCC

**Fig. 4:**
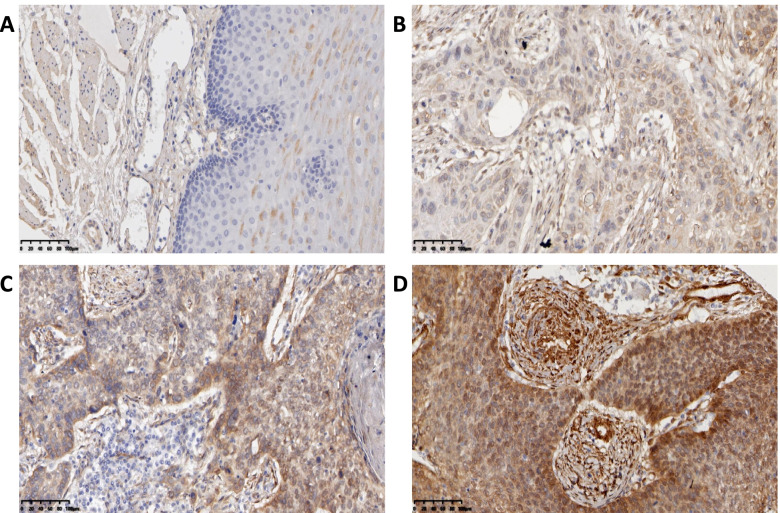
Immunohistochemical results of clinical ESCC paraffin specimens. **A** Expression of TAZ was negative in the adjacent tissues of normal esophageal carcinoma; **B** TAZ was low expressed in ESCC; **C** TAZ was moderately expressed in ESCC; **D** TAZ is highly expressed in ESCC

**Fig. 5:**
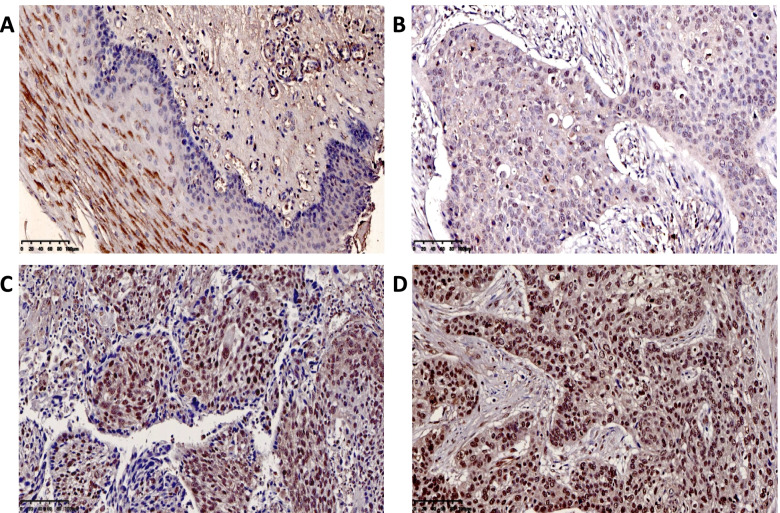
Immunohistochemical results of clinical ESCC paraffin specimens. **A** Expression of CTGF was negative in the adjacent tissues of normal esophageal carcinoma; **B** CTGF was low expressed in ESCC; **C** CTGF was moderately expressed in ESCC; **D** CTGF is highly expressed in ESCC

**Fig. 6:**
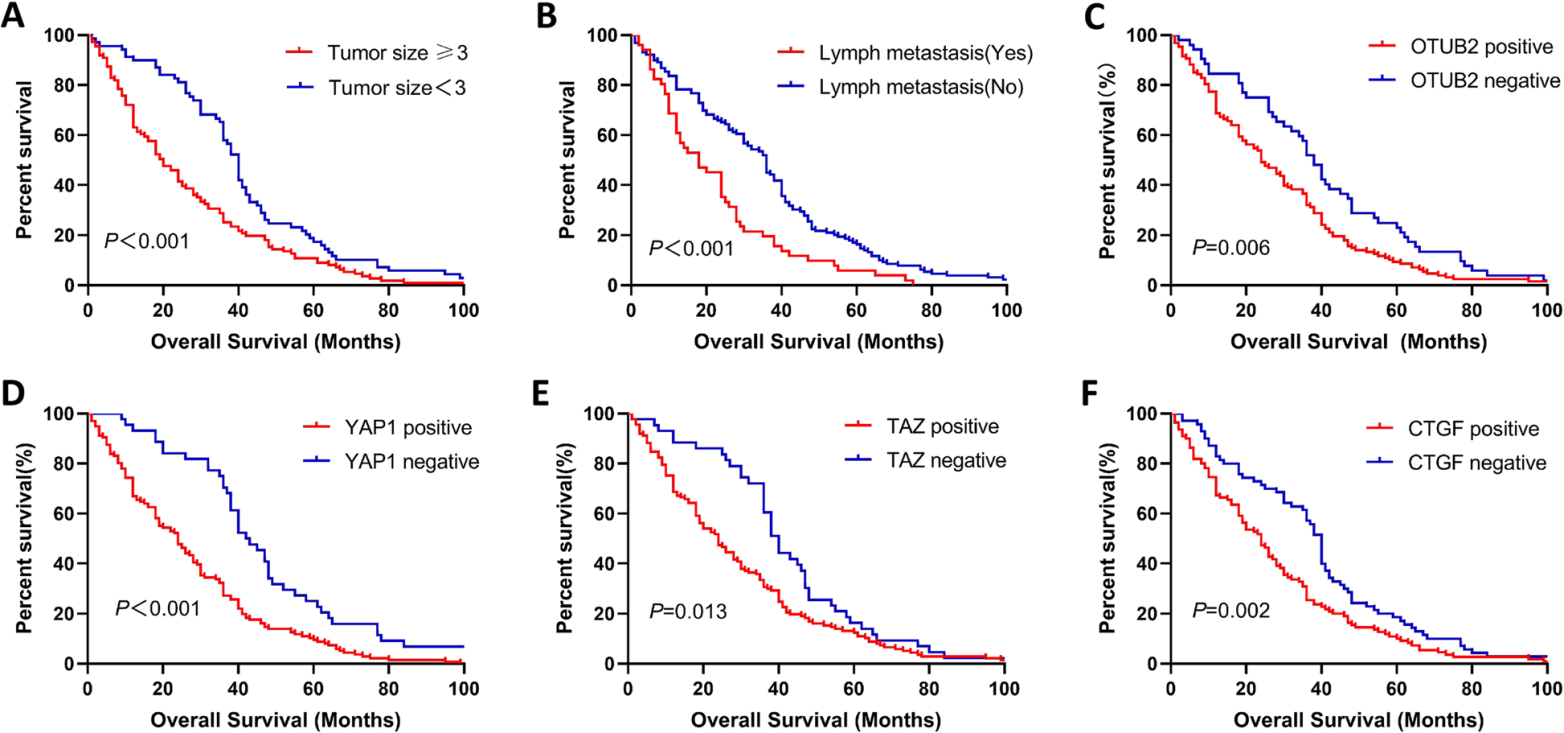
Kaplan-Meier survival curves for relevant parameters in patients with ESCC

Immunohistochemistry experiments analyzed 183 ESCC tissues and found that the expression of OTUB2, YAP1, TAZ and CTGF was significantly higher in ESCC than adjacent tissues, and the difference was statistically significant (*P* < 0.001) (Table 1). The expression of OTUB2 was negative in paracancerous tissues (Fig. 2A), but was highly expressed in the cytoplasm of ESCC (Fig. 2B-D); YAP1 and TAZ were negatively expressed in paracancerous tissues (Fig. 3A; Fig. 4A), but was highly expressed in the cytoplasm and was mainly located nucleus of ESCC (Fig. 3B-D; Fig. 4B-D). CTGF was negatively expressed in paracancerous tissues (Fig. 5A) and positively expressed in ESCC (Fig. 5B-D). After analysis of the relevant clinicopathological parameters, we found that the positive expression of OTUB2 was associated with tumor size (*P* = 0.003) and degree of differentiation (*P* = 0.037); The positive expression of YAP1 was mainly associated with tumor size (*P* = 0.011), degree of differentiation (*P* = 0.037), pathological stage (*P* = 0.027), and lymph node metastasis (*P* = 0.001); The positive expression of TAZ was mainly associated with tumor size (*P* < 0.001), infiltration depth (*P* = 0.020), pathological stage (*P* < 0.001) and lymph node metastasis (*P* = 0.001) (Table 2). The positive expression of CTGF was mainly associated with tumor size (*P* = 0.001) and lymph node metastasis (*P* = 0.008) (Table 2). To analyze the correlation of OTUB2 and YAP1, TAZ, CTGF protein expression in ESCC tissues, we found the positive expression of both OTUB2 and YAP1 was detected in 112 cases. Spearman correlation analysis revealed that the expression of OTUB2 was positively correlated with that of YAP1 (*r* = 0.374, *P* < 0.001) (Table 3); the positive expression of both OTUB2 and TAZ was detected in 109 cases. Spearman correlation analysis revealed that the expression of OTUB2 was positively correlated with that of TAZ (*r* = 0.271, *P* < 0.001) (Table 3); the positive expression of both OTUB2 and CTGF was detected in 89 cases. Spearman correlation analysis revealed that the expression of OTUB2 was positively correlated with that of CTGF (*r* = 0.216 *P* = 0.003) (Table 3). Kaplan-Meier survival curve indicated that when the tumor diameter was ≥3 cm, patients with lymph node metastasis had lower OS values (Fig. 6A, B). Further analysis showed that when the OTUB2, YAP1, TAZ and CTGF protein expression level was high, patients had a shorter OS(*P* < 0.05) (Fig. 6C-F). Among them, the OS of patients with tumor diameter ≥ 3 cm was 20 months, and the OS of patients with tumor diameter of < 3 cm was 40 months (*P* < 0.001); The OS of patients with lymph node metastasis was 18 months, and the OS of patients without lymph node metastasis was 36 months (*P* < 0.001);The OS was 24 months in the high OTUB2 expression group and 38 months in the low expression group (*P* = 0.006); The OS was 24 months in the high YAP1 expression group and 42.5 months in the low expression group; The OS was 24 months in the high TAZ expression group and 40 months in the low expression group (*P* = 0.013); The OS was 24 months in the high CTGF expression group and 40 months in the low expression group (*P* = 0.002); It suggested that patients with tumor diameter ≥ 3 cm, lymph node metastasis and high expression of OTUB2, YAP1, TAZ and CTGF protein had a poor prognosis.

**Table 1:** Expression of OTUB2, YAP1, TAZ, and CTGF in ESCC and adjacent tissues

Name		Pathological parameters/ N（%）		*χ*^*2*^value	*P *value
		ESCC tissue	Paracancerous tissue		
OTUB2	positive	130 (71.0)	49 (26.8)	71.739	< 0.001
	negative	53 (28.9)	134 (73.2)		
YAP1	positive	139 (75.9)	32 (17.5)	125.666	< 0.001
	negative	44 (24.1)	151 (82.5)		
TAZ	positive	140 (76.5)	35 (19.1)	120.723	< 0.001
	negative	43 (23.5)	148 (80.9)		
CTGF	positive	113 (61.7)	65 (35.5)	25.199	< 0.001
	negative	70 (38.2)	118 (64.5)		

*P*＜0.05, the difference was statistically significant

**Table 2:** Relationship between expression of OTUB2, YAP1, TAZ and CTGF in ESCC and clinicopathological parameters

Clinicopathological parameters	N	OTUB2 expression		*P*	YAP1 expression		*P*	TAZ expression		*P*	CTGF expression		*P*
	183	negative	positive		negative	positive		negative	positive		negative	positive	
Gender													
Female	54	11	43	0.097	15	39	0.444	11	43	0.519	20	34	0.827
Male	129	42	87		29	100		32	97		50	79	
Age													
< 60	67	22	45	0.380	18	49	0.497	13	54	0.321	24	43	0.607
≥ 60	116	31	85		26	90		30	86		46	70	
Tumor size													
<3	70	29	41	0.003	24	46	0.011	27	43	< 0.001	33	37	0.001
≥ 3	113	24	89		20	93		16	97		33	80	
Degree of differentiation													
Well differentiated	67	27	40	0.037	22	45	0.037	19	48	0.128	27	40	0.712
Moderately differentiated	98	22	76		21	77		23	75		35	63	
Poorly differentiated	18	4	14		1	17		1	17		8	10	
Depth of invasion													
Mucosa	15	2	13	0.250	6	9	0.134	7	8	0.020	6	9	0.910
Muscularis	90	30	60		24	66		24	66		33	57	
Full-thickness	78	21	57		14	64		12	66		31	47	
pTNM stage													
IB	49	17	32	0.576	18	31	0.027	22	27	< 0.001	25	24	0.087
IIA,B	113	30	83		24	89		20	93		39	74	
IIIA,B,C	21	6	15		2	19		1	20		6	15	
Lymph node metastasis													
No	131	43	88	0.067	40	91	0.001	39	92	0.001	58	73	0.008
Yes	52	10	42		4	48		4	48		12	40	

*P*＜0.05, the difference was statistically significant

**Table 3:** Correlation analysis of OTUB2 with YAP1, TAZ and CTGF

Name		OTUB2		*r *value	*P *value
		positive	negative		
YAP1	positive	112	27	0.374	< 0.001
	negative	18	26		
TAZ	positive	109	31	0.271	< 0.001
	negative	21	22		
CTGF	positive	89	24	0.216	0.003
	negative	41	29

### OTUB2 is Upregulated in ESCC Cell Lines

**Fig. 7:**
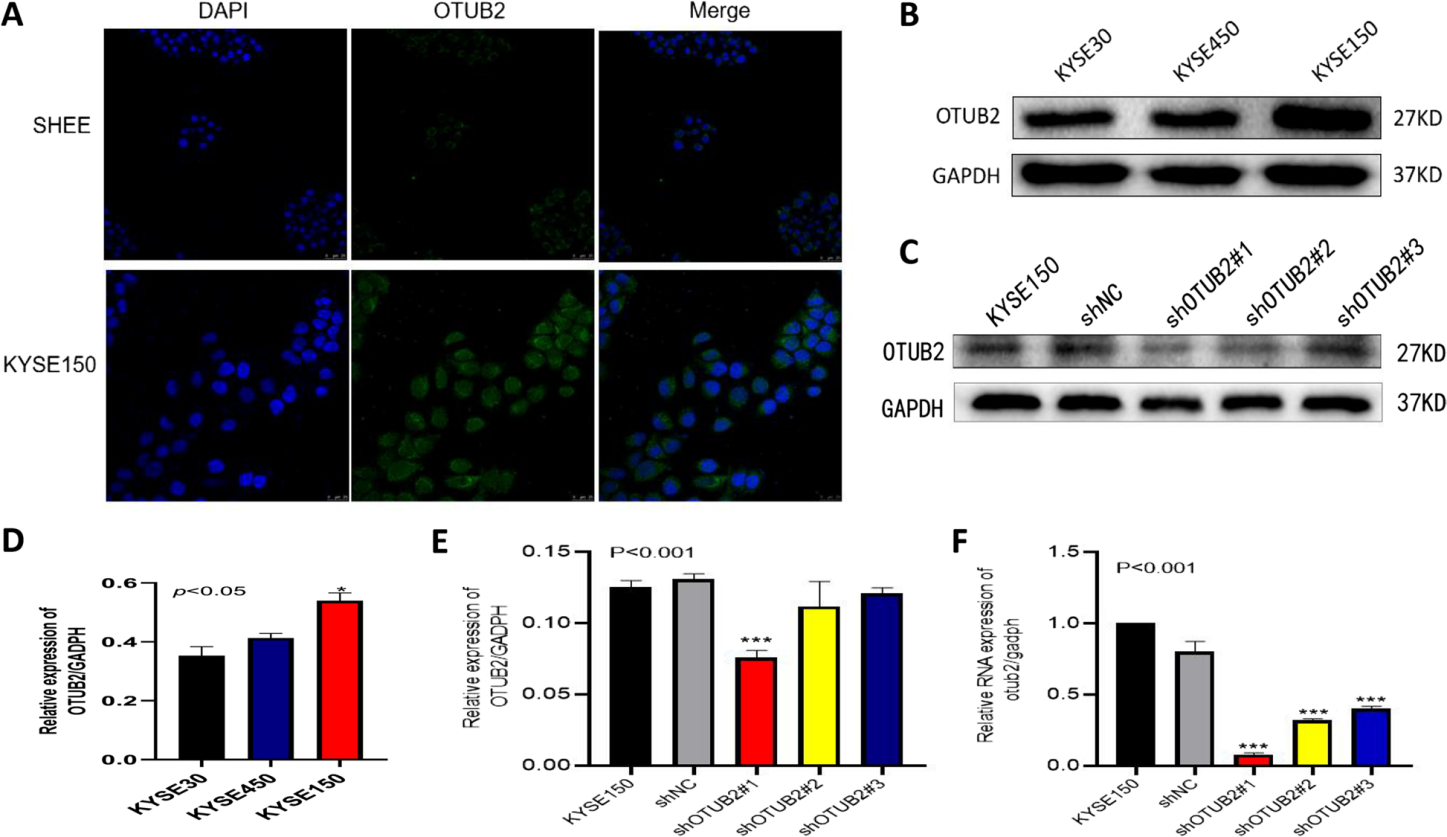
OTUB2 is upregulated in ESCC cell lines. **A** The expression of OTUB2 in SHEE and KYSE150 detected by immunofluorescence; **B** Western blotting showing OTUB2 expression in three esophageal squamous cell carcinoma cell lines; **C**&**F** Western blotting and qRT-PCR showing lentivirus transfection efficiency; **D**&**E** indicate the statistical comparisons of B and C respectively

On the basis of expression of OTUB2 in clinical tissues, we next verified the expression of OTUB2 at the cellular level. Immunofluorescence was used to detect the expression of OTUB2 in the normal esophageal epithelial cell line SHEE and the esophageal squamous cell carcinoma cell line KYSE150. The results showed that OTUB2 was slightly expressed in SHEE cells, and was mainly located in the cytoplasm, while in ESCC tissues, OTUB2 was strongly expressed in the cytoplasm, and there was a some expression in the nucleus, which was consistent with the expression and immunohistochemical results retrieved from the online databases (Fig. 7A). Western blotting was used to detect the expression of OTUB2 protein in three esophageal squamous cell carcinoma cell lines, and the cell line with the highest expression of OTUB2 was selected for lentiviral transfection to knockdown OTUB2 expression. The results showed that compared with KYSE30 and KYSE450 cell lines, the expression of OTUB2 was the highest in KYSE150 cells (Fig. 7B,D). Next, we used western blotting and qRT-PCR to screen the viral sequence with the highest knockdown efficiency. The results showed that compared with the normal control group, the expression of OTUB2 was eliminated best using the shOTUB2#1 vector (Fig. 7C,E,F). Therefore, we selected shOTUB2#1 to carry out the following cell function experiments.

### OTUB2 Knockdown Plays a Tumor Suppressing Role in ESCC Cells In Vitro

**Fig. 8:**
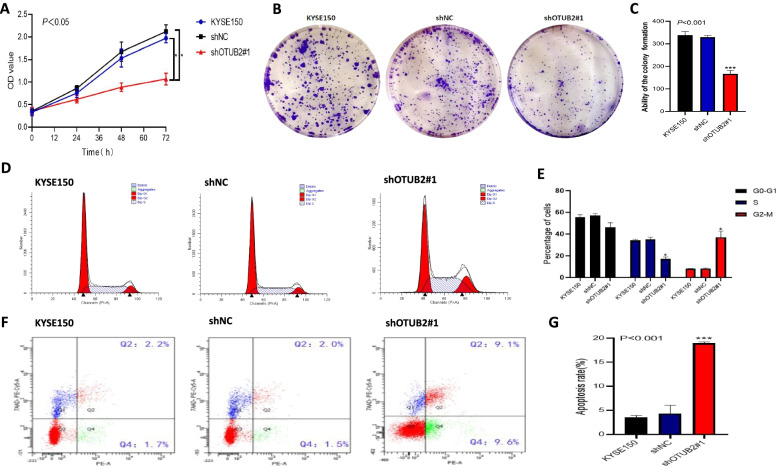
OTUB2 knockdown inhibits tumor proliferation in ESCC cells in vitro. **A** CCK-8 assay was used to detect cell proliferation; **B, C** Plate cloning experiment; **D, E** Cell Cycle Detection by Flow cytometry; **F, G** Apoptosis was detected by flow cytometry

**Fig. 9:**
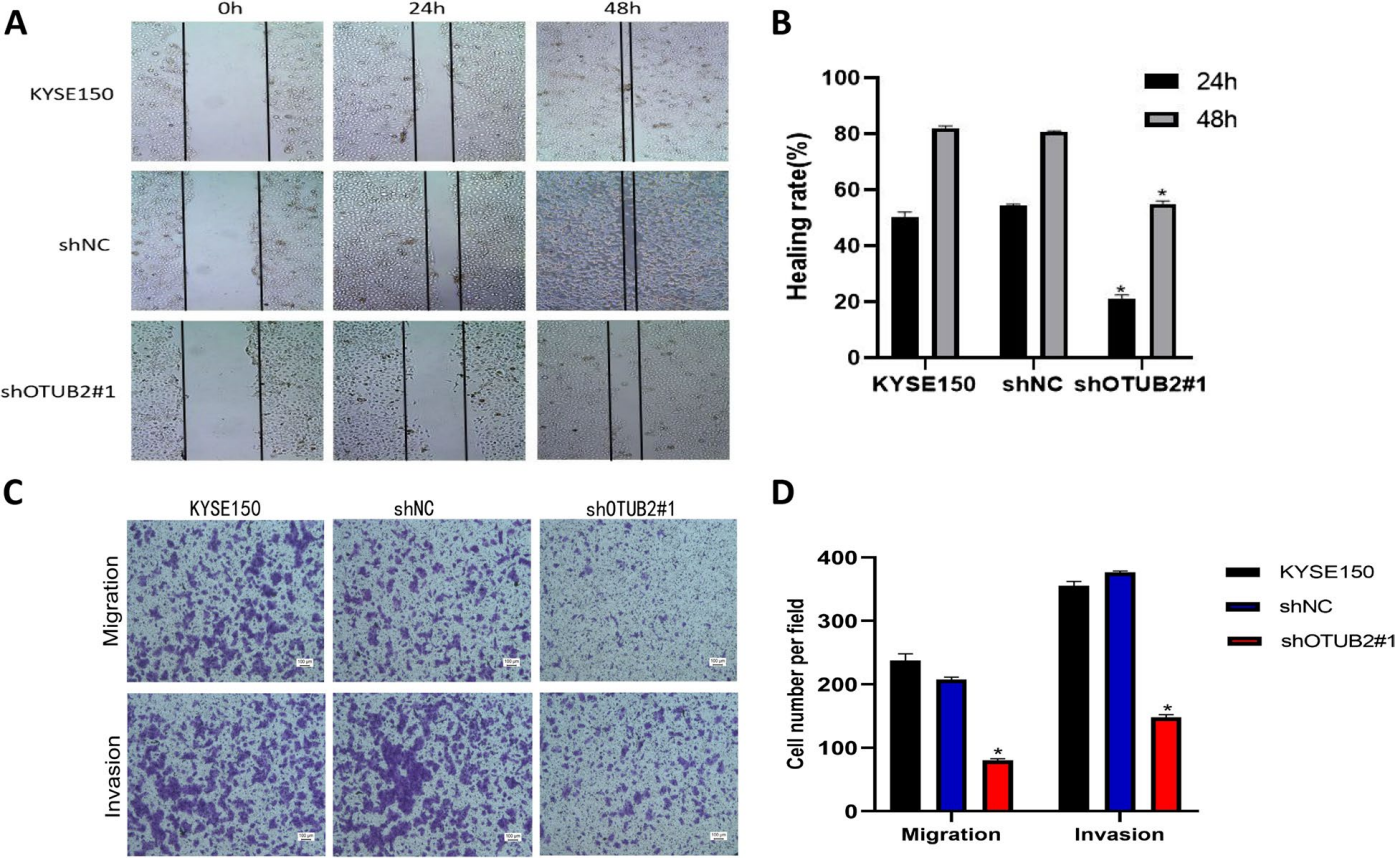
OTUB2 knockdown inhibits tumor migration and invasion in ESCC cells in vitro; **A, B**:Scratch healing test; **C-D** Transwell was used to detect cell migration and invasion

To observe the effects of OTUB2 knockdown on the proliferation, migration, and invasion of esophageal squamous cell carcinoma cells, we carried out cell function experiments. The CCK-8 proliferation assay showed that the proliferation ability of shOTUB2#1 cells was significantly lower than that of KYSE150 group and the shNC group (Fig. 8A). The plate clone formation assay showed that compared with the control group, the clone formation ability of KYSE150 and shNC was significantly decreased and the number of cells was decreased (*P* < 0.05), which suggested that the proliferation of ESCC could be inhibited by OTUB2 knockdown (Fig. 8B, C). The cell cycle was detected by flow cytometry. The results showed that the cells in shOTUB2#1 group were significantly blocked in G2/M phase, and the proportion of cells in S phase decreased. The difference was statistically significant(*P* < 0.05) (Fig. 8D, E); Flow cytometry was used to detect the apoptosis of three groups of cells. Compared with the control group, the apoptosis rate of shOTUB2#1 cells was significantly higher than that of ESCC cells (Fig. 8F, G). The results of the wound healing experiment showed that there was no significant difference in the width and area of wound healing between KYSE150 and shNC, but in KYSE150 cell line, the width and area of wound healing in shOTUB2#1 group were larger than those in controls, and the healing rate was lower, indicating a down-regulation of OTUB2 expression in KYSE150 can reduce the migration ability of tumor cells (Fig. 9A, B). In addition, we used Transwell chamber assays to evaluate cell migration and invasion. Compared with KYSE150 and shNC group, the number of migrated and invaded cells transfected with shOTUB2#1 cells decreased, and the migration and invasion ability decreased significantly (*P* < 0.05) (Fig. 9C, D). These results suggest that when the expression of OTUB2 in ESCC cell line KYSE150 was down regulated, the proliferation, migration and invasion of KYSE150 cells is inhibited, which further indicates that OTUB2 knockdown can inhibit the growth and progression of tumor.

### OTUB2 Regulates YAP1/TAZ in ESCC

**Fig. 10:**
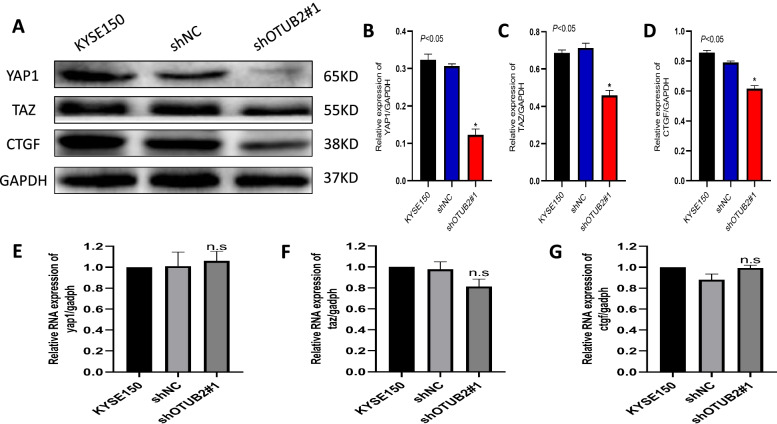
OTUB2 regulates YAP1/TAZ in ESCC. **A–D** Western blotting showing the expression of OTUB2, YAP1, TAZ and CTGF protein; **E–G** qRT-PCR showing the expression of the OTUB2, YAP1, TAZ and CTGF mRNA

In order to investigate whether OTUB2 regulates the expression of YAP1/TAZ, we used western blotting and qRT-PCR to detect the expression of OTUB2, YAP1, TAZ and CTGF in lentiviral interference knockdown cell line shOTUB2#1, KYES150, and shNC cells at the protein and mRNA levels. The results showed that the protein expression levels of YAP1, TAZ and CTGF were significantly decreased after OTUB2 knockdown in ESCC cell lines (*P* < 0.001);but the mRNA expression levels of YAP1, TAZ and CTGF were not significantly decreased after OTUB2 knockdown in ESCC cell lines (Fig. 10). This phenomenon indicated that OTUB2 may promoting YAP1 and TAZ protein expression during the development of ESCC, and the role of OTUB2 in promoting the development of ESCC may mainly occur via YAP1 and TAZ.

## Discussion

The incidence and mortality rate for esophageal cancer are the highest in the world. Its malignancy, invasion, and metastasis are usually closely related to the proliferation and differentiation ability of the cancer cells. In addition, a variety of internal and external environmental factors can affect the occurrence of esophageal cancer, such as exposure to alcohol and tobacco, esophageal injury, esophageal disease, and food including, nitrosamine compounds, mycotoxins, and genetic factors may be causes of esophageal cancer, but a specific cause for esophageal cancer is not clear [1]. Therefore, exploring the possible pathogenesis of ESCC is of great significance for early and accurate diagnosis of ESCC and to improve the overall survival rate of ESCC patients.

OTUB2, or otubain 2, is a deubiquitinase, a member of the protein superfamily of ovarian tumor (OTU) was identified as a Drosophila ovary tumor gene for the first time. The OTU domain in OTUB2 has been identified in numerous mammals and encodes about 130 amino acids. The OTUs protease family consists primarily of a group of hypothetical cysteine proteases such as A20, OTUB1, OTUB2, and yeast OTUB1 [15, 16]. The members of this family are specific for identifying polyubiquitinated chains with different junction types. There are several kinds of deubiquitinating enzymes (DUBs) with diverse structures and complex functions. As the key regulatory factors of protein ubiquitination, they may directly influence the activity, regeneration, and localization of a variety of proteins in cells. In addition, DUBs play an important role in the regulation of cell apoptosis, the cell cycle, and DNA damage repair, and are closely associated with the occurrence and evolution of tumors [17–19]. OTUB2 can be used as a tumor stem cell and metastasis promoting factor, and is associated with the progression of different tumors.

YAP1 and TAZ are core downstream effectors of the Hippo signaling pathway, which play a role in cell adhesion, proliferation, migration, and cell fate determination [20]. The main mechanisms in tumor development involve YAP1/TAZ. YAP1/TAZ is highly conservative during normal cell growth, and is located mainly in the cytoplasm in the phosphorylated form. They participate in cell signaling and in the regulation of transcriptional activities of different transcription factors; In pathological states, some upstream factors weaken the inhibition of YAP1/TAZ and enhance their own activity, which results in decreased phosphorylation and enhanced dephosphorylation. They translocate from the cytoplasm to the nucleus and bind with the nuclear transcription factor TEAD_1–4_, thus participating in tumor proliferation, invasion, and metastasis [21, 22]. A significant amount of data show that YAP1/TAZ exhibits abnormally high activity in a variety of tumors, and is closely associated with the migration and invasion ability of tumors.

OTUB2 is polysumoylated at position lysine 233, and there may be a SUMO, or small ubiquitin-related modifier, interacting motif (SIM) in YAP1/TAZ, which may play an important role in its interaction combining this domain with sumoylated OTUB2. YAP1/TAZ activity is mainly affected by various factors, including its transcriptional activity, protein stability and nucleoplasmic shuttling, while deubiquitylase can directly affect the protein stability of YAP/TAZ through its ubiquitination effect, and its regulation of YAP/TAZ is a post-transcriptional modification. Zhang et al. [5], from Zhejiang University, determined that OTUB2 may act as an important modulator to regulate the expression of YAP1/TAZ, thus regulating the occurrence and development of tumor. OTUB2 can bind and stabilize YAP1/TAZ through ubiquitination, and has a certain positive regulatory effect on YAP1/TAZ. These authors found that OTUB2 could directly act on YAP1/TAZ independently of the Hippo signaling pathway to promote tumor proliferation. When OTUB2 is overexpressed, it can directly stabilize and bind to YAP1/TAZ through SUMO, activate the YAP1/TAZ expression and activating downstream target genes such as CTGF and CYR61 to promote the proliferation, invasion and metastasis of tumor cells. They also found that OTUB2 may be an important upstream regulator of YAP/TAZ expression, regulating the development of tumors by affecting the protein expression of YAP/TAZ, which has almost no effect on YAP/TAZ mRNA levels.

It has also been reported that OTUB2 promotes homologous recombination repair of endometrial carcinoma through YAP/TAZ mediated Rad51 expression, which provides a potential therapeutic target for endometrial carcinoma. Silencing of OTUB2 can inhibit the growth of endometrial cancer and increases the sensitivity of tumor to anti-tumor drugs [8]. In the HCC cell line, knocking-down OTUB2 expression can significantly inhibit the growth of liver cancer cells [9]. All the above studies suggest that OTUB2 may play a role in promoting tumorigenesis. However, the specific mechanisms involved in OTUB2 and YAP1/TAZ in ESCC still need to be further studied.

This study first analyzed the expression of OTUB2 in esophageal squamous carcinoma and its relationship with YAP1/TAZ via bioinformatics analysis, and combined with immunohistochemical staining of paraffin tissue samples from ESCC patients and their associated clinicopathological parameters, we explored the expression of OTUB2 and YAP1/TAZ in ESCC and their relationship with relevant clinical parameters. Secondly, the expression of OTUB2 in ESCC cell lines and its role in ESCC cell proliferation, invasion and metastasis was evaluated at the cellular and molecular levels. We determined that OTUB2 was highly expressed in ESCC tissues and cell lines. When we silenced OTUB2 using lentivirus interference in an ESCC cell line, the progression of ESCC was significantly inhibited. Additionally, we also determined that the expression of YAP1,TAZ and CTGF protein also decreased significantly with the silencing of OTUB2, but the mRNA were not significantly changed, this result is also consistent with the findings in breast cancer by Zhang Z [5] et al.,which suggested that the effect of OTUB2 on YAP1/TAZ may not be regulated by transcriptional level, but by post-transcriptional modification of YAP1/TAZ through its deubiquitinating enzyme action, affecting the protein level of YAP1/TAZ and then exerting synergistic tumor-promoting effects.

The limitations of this study are that we have only provided preliminary data and interpretation based on clinical tissue samples, and in vitro assays at the cellular and molecular levels. Our group will also carry out further research and exploration using in vivo animal models. The results of this study are expected to provide a new theoretical basis for the early detection, diagnosis, and treatment of ESCC, and may provide a new target for the clinical treatment of ESCC.

## Supplementary Information

**Additional file 1.**

## Data Availability

The datasets supporting the conclusion of this article are included within the article.

## Abbreviations

ESCC: Esophageal Squamous Cell Carcinoma
OTUB2: Otubain 2
OTU: Ovarian Tumor Domain
YAP1: Yes Related Protein 1,
TAZ: Transcriptional Coactivator with PDZ binding motif,
CTGF: Connective Tissue Growth Factor
TEADs: TEA domain DNA-binding family of transcription factors.
DUBs: Deubiquitinating Enzymes
SUMO: Small Ubiquitin-related Modifier
