# Supplementary material for: OTUB2 Regulates YAP1/TAZ to Promotes the Progression of Esophageal Squamous Cell Carcinoma
Source: Biol Proced Online. 2022 Jul 18;24:10. doi: 10.1186/s12575-022-00169-9 (PMC9290284; doi:10.1186/s12575-022-00169-9)

Western blotting images trimming of the corresponding target protein was trimmed according to the location of marker.The original blot images used in the article are specific as follows：

Western blotting showing OTUB2 expression in three esophageal squamous cell carcinoma cell lines(KYSE30; KYSE450; KYSE150)--OTUB2,molecular weight:27KD; GAPDH,molecular weight:37KD

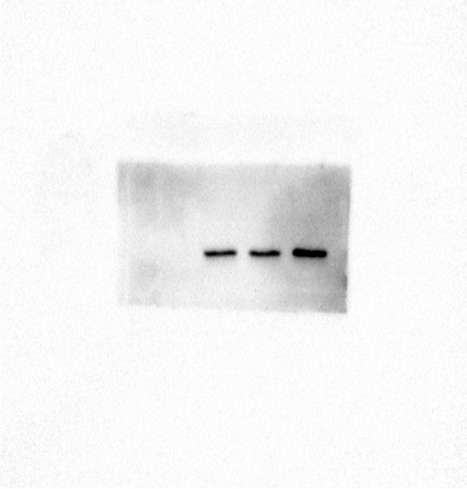

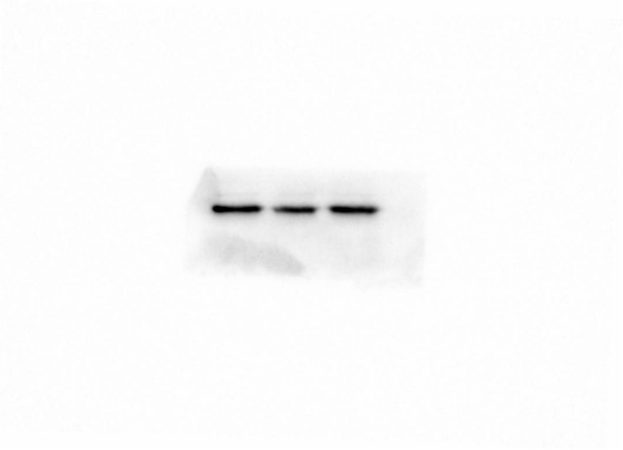

Western blotting showing lentivirus transfection efficiency (KYSE150; shNC; shOTUB2#1; shOTUB2#2; shOTUB2#3)--OTUB2, molecular weight:27KD; GAPDH,molecular weight:37KD

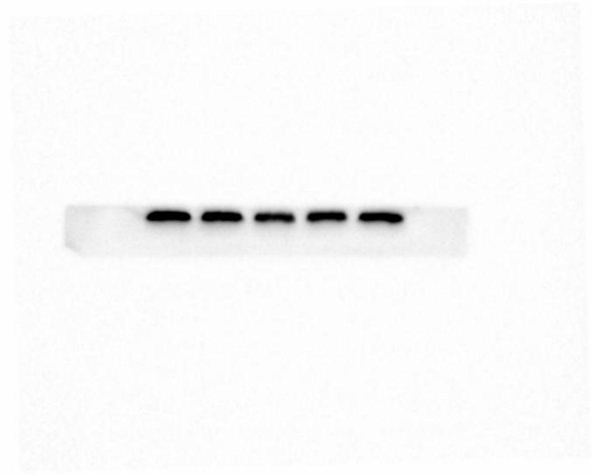

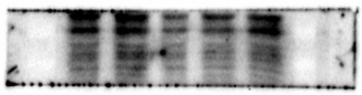

Western blotting showing the expression of YAP1, TAZ and CTGF protein after OTUB2 knockdown (KYSE150; shNC; shOTUB2#1) GAPDH,molecular weight:37KD; YAP1,molecular weight:65KD; TAZ,molecular weight:55KD; CTGF,molecular weight:38KD.

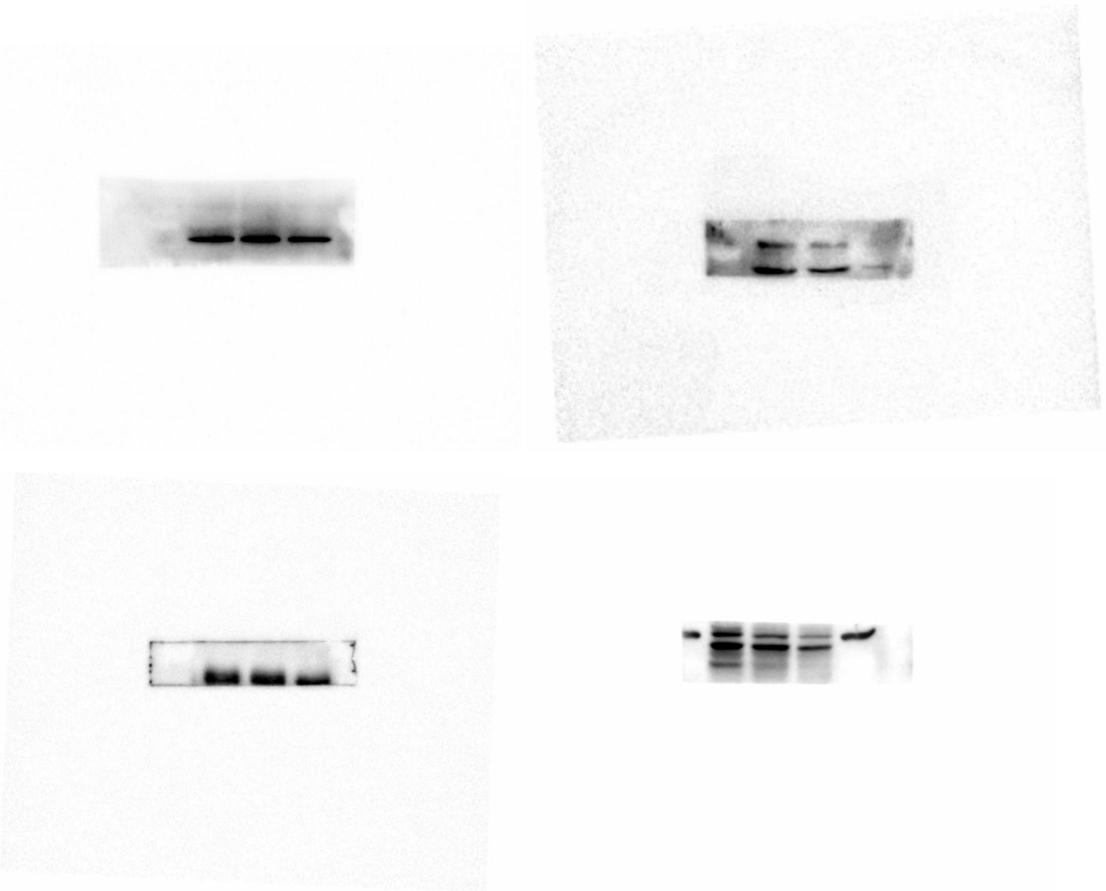

Supplement: Supplementary file 1 — Additional file 1. [file 12575_2022_169_MOESM1_ESM.zip › Supplementary/Original blot images-Western blot.pdf]
